# A mouse model of immunosuppression facilitates oral *Candida albicans* biofilms, bacterial dysbiosis and dissemination of infection

**DOI:** 10.3389/fcimb.2024.1467896

**Published:** 2025-01-20

**Authors:** Raja Veerapandian, Anuja Paudyal, Sarah M. Schneider, Sonny T. M. Lee, Govindsamy Vediyappan

**Affiliations:** ^1^ Division of Biology, Kansas State University, Manhattan, KS, United States; ^2^ Diagnostic Medicine and Pathobiology, Kansas State University, Manhattan, KS, United States

**Keywords:** oropharyngeal candidiasis, *Candida albicans*, mice model, immunosuppression, gut microbiome, *Enterococcus*, bacterial dysbiosis, dissemination of infection

## Abstract

Opportunistic pathogens are a major threat to people, especially those with impaired immune systems. Two of the most important microbes in this category are the fungus *Candida albicans* and Gram-positive bacteria of the genus *Enterococcus*, which share overlapping niches in the oral cavity, gastrointestinal and urogenital tracts. The clinical importance of oral *C. albicans* biofilm and its interaction with the host under immunosuppressive conditions remains largely understudied. Here, we used a mouse model of oropharyngeal candidiasis (OPC) with cortisone acetate injection on alternate days and a continuous supply of *C. albicans* in drinking water for three days, resulting in immunosuppression. Results showed abundant growth of resident oral bacteria and a strong *C. albicans* biofilm on the tongue consisting of hyphae which damaged papillae, the epidermal layer, and invaded tongue tissue with the accumulation of inflammatory cells as demonstrated by Grocott’s methenamine silver and hematoxylin and eosin staining, respectively. The dispersed microbes from the oral biofilm colonized the gastrointestinal (GI) tract and damaged its integrity, disseminating microbes to other organs. Although no visible damage was observed in the kidney and liver, except increased lipid vacuoles in the liver cells, *C. albicans* was found in the liver homogenate. Intriguingly, we found co-occurrence of *Enterococcus faecalis* in the tongue, liver, and stool of immunosuppressed control and *C. albicans* infected organs. Targeted 16S rRNA and ITS2 amplicon sequencing of microbes from the fecal samples of mice confirmed the above results in the stool samples and revealed an inverse correlation of beneficial microbes in the dysbiosis condition. Our study shows that mucosal-oral infection of *C. albicans* under immunosuppressed conditions causes tissue damage and invasion in local and distant organs; the invasion may be aided by the overgrowth of the resident endogenous Enterobacteriaceae and other members, including the opportunistic pathogen *Enterococcus faecalis*.

## 1 Introduction

Opportunistic pathogens pose a great challenge with significant mortality in people with an impaired immune system. Among them, *C. albicans* and *Enterococcus* species are important pathogens, which inhabit common niches in the normal mammalian microbiome (Oever and Netea, 2014; d’Enfert et al., 2021). These pathogens do not cause disease in healthy hosts but when there is a weakened immune system these organisms, especially *C. albicans*, can overgrow and cause life-threatening infections in patients with human immunodeficiency virus (HIV), Sjogren’s syndrome, diabetes mellitus, head and neck cancers, and coronavirus disease 2019 (COVID-19) (Sangeorzan et al., 1994; Rhodus et al., 1997; Redding et al., 1999; Willis et al., 1999; Chen et al., 2020; Riad et al., 2021). *C. albicans* is a causative agent of oropharyngeal candidiasis (OPC) where it proliferates in the mouth and the morphological switch from commensal yeast to invasive hyphae leads to severe oral thrush (biofilm) (Kong et al., 2015; Swidergall et al., 2021).

The interaction between *C. albicans* and the host is a dynamic one that can be altered, depending on host factors including changes in temperature and pH, the coexistence of other oral microbiome competing for nutrients and space, and host immune properties including, antimicrobial peptides (Jakubovics, 2015; Forche et al., 2018). This fungal pathogen uses a variety of strategies to adhere, adapt and proliferate in response to host environmental conditions. The gene expression pattern of *C. albicans* is extremely important in adaptation to different environmental conditions (i.e., differences in pH and nutrient level) (Forche et al., 2018).

In the immunosuppressed host, bacterial communities may alter leading to dysbiosis. When there is an imbalance in the bacterial community, polymicrobial interactions including those involving *C. albicans* will change. Under certain circumstances this change can facilitate infection of the host (Bertolini et al., 2019). There are some well-reported mouse models of OPC using corticosteroids as an immunosuppressant in mice (Kamai et al., 2001; Dwivedi et al., 2011; Solis and Filler, 2012; Xu et al., 2014; Kobayashi-Sakamoto et al., 2018). In the present study, in addition to cortisone acetate as immunosuppressant and oral swab infection, an infection of *C. albicans* in drinking water for the initial three days was used to mimic high oral fungal burden in immunosuppressed patients (e.g., HIV, those undergoing radiation therapy for head and neck cancer, etc.) with oral *Candida* present in their saliva passing through the GI tract by swallowing. There are few animal studies that have determined fungal growth and dissemination during immunosuppression, and typically these have included use of antibiotics or studied co-infection with other bacterial species (Xu et al., 2014; Kong et al., 2015; Kobayashi-Sakamoto et al., 2018).

*C. albicans* and *Enterococcus* species are opportunistic pathogens that can proliferate under immunosuppressed condition. Similar to *C. albicans*, enterococcal members cause life-threatening diseases in humans and serve as a source for spreading drug resistance (vancomycin) among other bacteria. For example, *E. faecalis* causes colonic anastomotic leak via its collagenase secretion, a dreaded complication in cancer surgery patients who normally undergo chemotherapeutic and immunosuppressive regimens (Shogan et al., 2015; Anderson et al., 2021). While *Enterococci* are primarily found in the GI tracts of animals and humans, and in the environment possibly by fecal contamination, they are also found in the oral cavities of animals and humans. *Enterococci* cause infections (bacteremia, implanted medical device infections, urinary tract infections, and endocarditis) in hospitalized patients. They are intrinsically resistant to several antibiotics and are the third leading cause of nosocomial infections in the U.S (Jett et al., 1994; Weiner et al., 2016; Fiore et al., 2019). Thus, understanding their pathogenesis and dissemination under immunosuppressed conditions is critical for their management.

The clinical importance of the fungal biofilm and its dissemination with endogenous flora under immunosuppression is poorly studied. To better understand the *in vivo* interaction between these endogenous bacteria and *C. albicans* without the influence of antimicrobial agents, the mouse infection model serves as an ideal platform since it recapitulates human infections. In the present study, we have demonstrated the formation of fungal biofilm in the oral cavity, and we monitored the dissemination of fungal and bacterial populations using histology, colony-forming unit (CFU), and microbiome analysis. The data help us to understand how immunosuppression and *C. albicans* oral infection can lead to microbial dysbiosis and their dissemination of infection. The present OPC model can also be useful in evaluating therapies to control OPC and determining the influence of endogenous bacteria on candidiasis.

## 2 Materials and methods

### 2.1 Ethics statement and animal care

All animal studies were conducted at the Comparative Medicine Group at the college of Veterinary Medicine, Kansas State University. Experiments had received prior approval from the Institutional Animal Care and Use Committee (IACUC) of Kansas State University (IACUC Protocol #4248.1). Animals were housed with unlimited access to water and mouse chow and permitted to move without restraints within their cages. All efforts were made to minimize any discomfort, distress, and pain to animals.

### 2.2 *C. albicans* strain and growth conditions

*C. albicans* SC5314, a genome sequenced laboratory strain originally isolated from a patient with bloodstream infection, has been widely used in oral biofilm mouse models (Solis and Filler, 2012; Bertolini et al., 2019). *C. albicans* was routinely maintained in yeast extract (1%) peptone (2%) dextrose (2%) (YPD) agar (1.5%) plate. For animal experiments, a single *C. albicans* colony, initiated from a glycerol stock, was suspended in YPD broth and grown overnight at 30°C with shaking. Growing *C. albicans* at 30°C allows it to produce mostly yeast cells, which is convenient for determining the infectious dose. *C. albicans* is known to produce multicellular hyphae at 37°C. Cells were harvested and washed twice with PBS, and final cell density was adjusted as required.

### 2.3 Cortisone acetate preparation and mouse model of oropharyngeal candidiasis

Cortisone acetate (Sigma-Aldrich, St. Louis, MO, USA) was suspended (225 mg/kg body weight) in sterile PBS without calcium and magnesium (Gibco) containing 0.05% (v/v) Tween 80 (Sigma-Aldrich, St. Louis, MO, USA). The prepared cortisone acetate solution was made as suspension by sonication in a water bath for 1 min using Branson sonicator. Before injection, the preparation was vortexed and injected immediately (Solis and Filler, 2012). To avoid lot-to-lot variation, a single batch of cortisone acetate was used.

Our OPC model was designed with some modifications from previously established oral mouse model protocols (Solis and Filler, 2012; Xu et al., 2014). Eight-week-old female C57BL/6 mice (Charles River Laboratories, Wilmington, MA, USA) were purchased and acclimated one-week to housing before initiating the experiment. Animals were divided into two groups with n=6/group: (1) Cortisone acetate alone control, (2) Cortisone acetate + *C. albicans* infection. Two independent experiments were performed. Briefly, mice were immunosuppressed on day -1 and on alternate days (day 1 and 3) with cortisone acetate (subcutaneously) dissolved in 0.05% Tween 80/PBS. On day 0, mice were anesthetized by an intraperitoneal injection of ketamine: xylazine (100 and 10 mg/kg of body weight, respectively). Then the oral cavity was swabbed with a sterile cotton swab soaked with 100 µl of *C. albicans* (6 × 10^8^ cells/mL) or PBS which was left sublingually for 1 h. Fresh suspensions of *C. albicans* (6 × 10^6^ yeast cells/mL) were added to the drinking water for 3 days (day 0 to 2), to maintain high oral carriage loads (Bertolini et al., 2019). Microbes in drinking water were changed every 24 h. After 2 days, normal drinking water was supplied until the end of the experiment. To ensure the viability of the yeast in the drinking water, the water was sampled and cultured on a YPD plate after 24 h, and the results confirmed the survival of yeast (data not shown). On day 5, all mice were sacrificed and the organs, including tongue, intestine, kidney, and liver were excised and processed for analysis.

### 2.4 Tongue pathological scoring

All tongues were excised and digitally photographed for biofilm analysis. Image analysis was done by ImageJ software (National Institute of Health, Bethesda, MD, USA). Tongue scoring (0-4) was done based on the percentage of the surface area affected as published elsewhere (Dwivedi et al., 2011; Xu et al., 2014) with slight modifications. Tongues with no lesions = 0, unmeasurable lesions = 1, measurable lesions covering < 25% surface area = 2, between 25–75% area = 3, and > 75% surface area = 4.

### 2.5 Determination of CFU and 16S rRNA gene PCR

For CFU determinations, tongues, kidneys, and livers from control and experimental animals were excised, weighed, and homogenized. To calculate the fungal burden, homogenized tissue aliquots were plated on YPD agar containing chloramphenicol (10 μg/mL, Sigma) and kanamycin (50 μg/mL, Sigma). For *Enterococcus* sp. CFU determination, samples were plated on bile esculin (BE; Difco Bile Esculin Agar, Becton, Dickinson and Company (BD), Franklin Lakes, NJ, USA) agar and black colored colonies were counted. When necessary, colonies were plated or re-streaked on BHI (brain heart infusion agar, BD) containing 6.5% NaCl to further confirm the identification of *Enterococci*. On BHI salt-agar, the *Enterococci*, but not the viridian-D *Streptococci* (e.g., *S. bovis*) are expected to grow. Fresh stool samples were collected from each mouse, weighed, and homogenized in PBS. CFU for homogenized stool samples were determined on BHI, BHI + NaCl or BE agar plates. For oral microbe determination after immunosuppression, oral swabs were taken before and after the cortisone injection and plated on BHI or BE agar plates. Oral sampling was done for 30 s as previously described (Abusleme et al., 2017). Briefly, a sterile cotton swab was used to swab the oral cavity, starting from the tongue, then buccal areas, gingiva, palate, and ending with the gingiva on the lower incisors.

To differentiate between *E. faecalis* and *E. faecium* from mice oral samples, the 16S rRNA gene was amplified by PCR using genomic DNA and species-specific 16S rRNA primers (Ryu et al., 2013). The PCR primers for *E. faecalis* (forward 5-CATAACAGTTTATGCCGCATGGCATAAGAG-3, reverse 5-GGGGACGTTCAGTTACTAACG-3, accession number DQ239694) and *E. faecium* 16S rRNA (forward 5-TATAACAATCGAAACCGCATGGTTTTGATT-3, reverse 5- AGGGATGAACAGTTACTCTCA-3, accession number AY675247.1) genes were employed, and the amplified PCR products were sequenced commercially by the Sanger DNA sequencing method. The DNA sequences were analyzed using the NCBI nucleotide BLAST program under default mode. Sequences with the highest similarities (>70%) were considered matches. The 16S rRNA DNA sequence obtained in this study was deposited in the GenBank with accession number MZ868593.

### 2.6 Metagenomic sequence of 16S and ITS amplicons

To determine the changes in the gut bacterial and fungal communities of the immunosuppressed *C. albicans* infected and uninfected mice, we took the targeted sequencing of the 16S rRNA gene and ITS2 amplicons for bacteria and fungi, respectively. Fecal samples from normal non-immunosuppressed and uninfected mice were also included as additional control. Fresh fecal samples collected from these three mice groups were stored at -80 ° C immediately after collection. Genomic DNAs were extracted using QIAmp PowerFecal isolation kit (Qiagen, Germantown, MD, USA), purified using Qiagen DNA purification kit, analyzed for quantity and quality by Qubit/Nanodrop and kept at -20°C until sequencing. The bacterial 16S rRNA gene V4 region was amplified during library preparation via Illumina’s Nextera XT Index Kit v2 (Illumina, Inc., San Diego, CA) (primers: 515F, 5-GTGCCAGCMGCCGCGGTAA-3 and 806R, 5-GGACTACHVGGGTWTCTAAT-3) (Caporaso et al., 2011). Similarly, the internal transcribed spacer region ITS2 was amplified using the primers fITS7/ITS4 combination (White et al., 1990; Ihrmark et al., 2012) (primers: 5-GTGARTCATCGAATCTTTG-3 & 5-TCCTCCGCTTATTGATATGC-3) (Sarkar et al., 2022). The quality of the libraries was confirmed by a bioanalyzer. Library preparation and subsequent sequencing also included a no-template negative control. Sequencing was performed on an Illumina MiSeq, which generated paired-end 250 bp reads at the Kansas State University’s Integrated Genomics Facility. DNA sequences were analyzed as described (Sarkar et al., 2022), and the taxonomic assignments of the microbes were visualized using QIIME 2 (Bolyen et al., 2019). The mice gut amplicon DNA sequence obtained in this study was deposited in the NCBI (National Center for Biotechnology Information) repository with accession number PRJNA1142050.

### 2.7 Calcofluor white staining for fungal cells

Fresh tissues samples were homogenized and smeared over the slide followed by methanol fixation for 5 min. The slide was rinsed with distilled water and stained with calcofluor white solution (0.1%) in PBS for 5 min. After two washes with distilled water, dehydration was done with 95% ethanol, absolute ethanol, and then xylene. Coverslips were placed and slides were imaged using a fluorescence microscope (Leica DM-6B).

### 2.8 Histopathology

Portions of tongue, liver, kidney, and cecum were collected and immediately placed in 10% buffered formalin for fixation. Mouse intestines were collected and processed with a gut bundling technique as published elsewhere (Williams et al., 2016). Fixed tissues were processed on a Sakura Tissue-TEK VIP 6 processor prior to paraffin embedding. Four micrometer thick serial sections were stained with hematoxylin and eosin (H & E), Grocott’s methenamine silver (GMS), or periodic acid Schiff’s (PAS) stains on a Leica Autostainer XL ST5010. An Olympus U-TV0.5XC-3 scope mounted camera using CellSens software acquired the slide images.

### 2.9 Statistical analysis

Data were analyzed for statistical differences using the Graph-Pad Prism 9.0 software. Statistical significance was determined by unpaired two-tailed t-test, assuming equal variances, or the Mann Whitney test when data were not normally distributed. Statistical significance for all tests was set at *P* < 0.05. *P*-values for each analysis are indicated in the representative graphs, where “ns” denotes non-significant. Each symbol represents one mouse or group.

## 3 Results

### 3.1 Oropharyngeal candidiasis infection model

**Figure 1:**
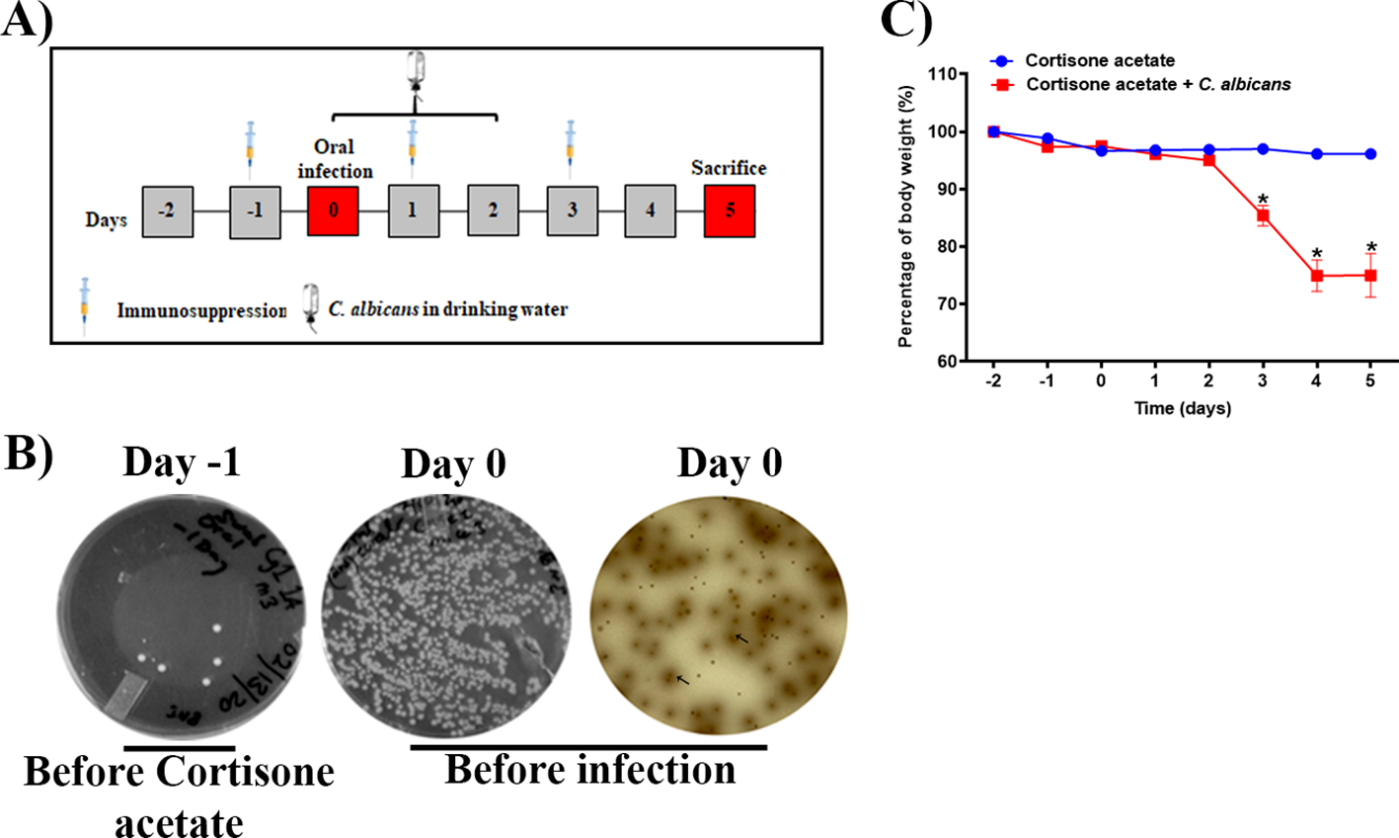
Oropharyngeal candidiasis (OPC) infection model. **(A)** A schematic of experimental design is shown. **(B)** An oral swab from a representative mouse taken on day -1 (left) and on day 0 (before infection) (middle) were streaked on BHI agar plates. The oral swab of day 0 was also streaked partially on bile esculin agar plate (right). The diffused-brown colored colonies (arrows) are indicative of *Enterococcus* species. **(C)** Body weight loss during the five-day experimental period expressed as a percentage of initial weight (day-2) in 6 animals per group from two independent experiments. Data were analyzed for statistical significance using the Graph-Pad Prism 9.0 software. Error bars represent SEM of n = 12 mice per time point. *P* values of <0.05 (*) were considered significant. Statistical significance was determined by unpaired two-tailed *t*-test.

**Figure 2:**
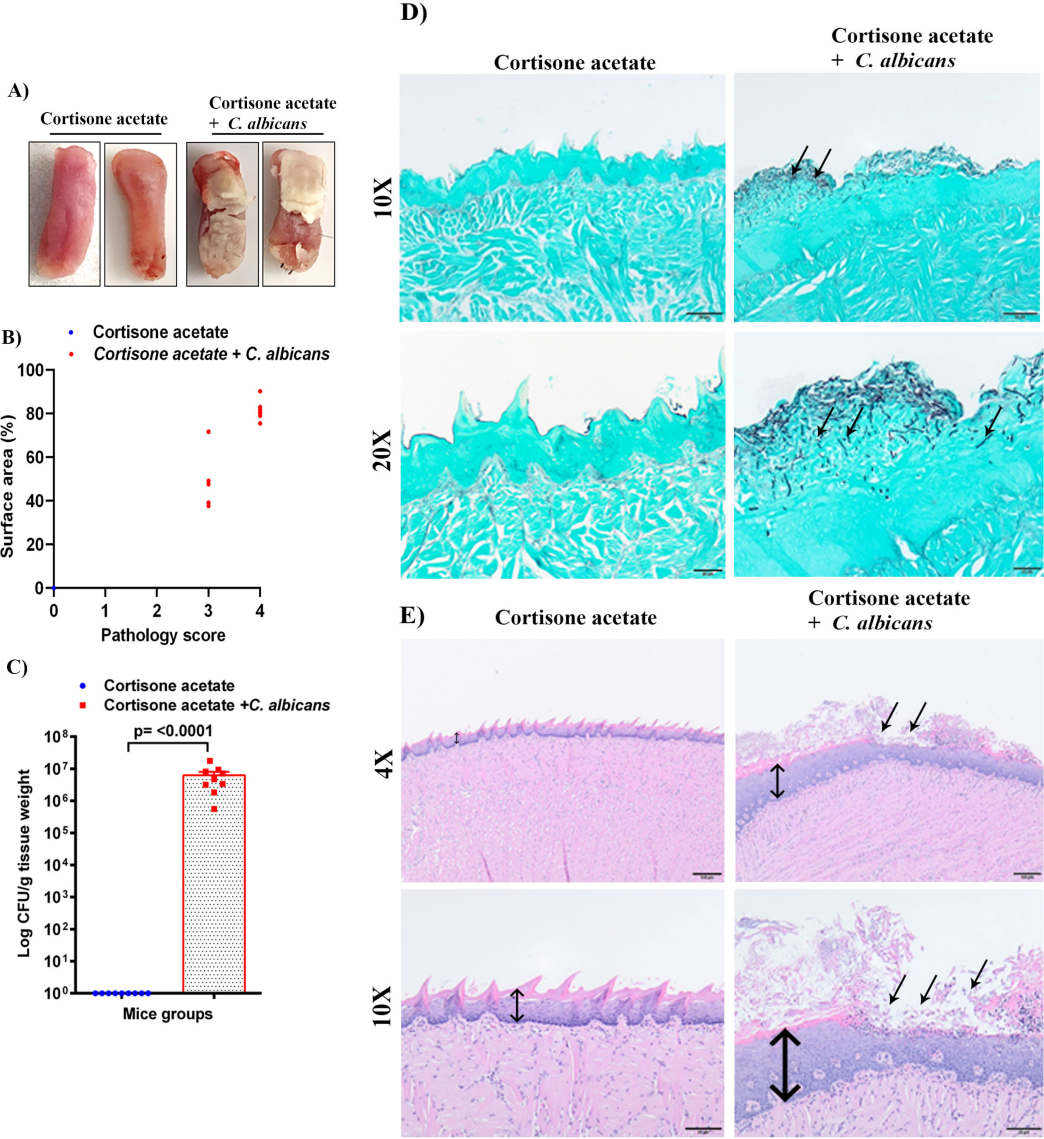
*C. albicans* forms biofilms in immunosuppressed mice. **(A)** Tongues of mice were excised after five days of infection and the dorsal aspect was digitally photographed. Representative pictures are shown from two mice in each group. **(B)** Oral pathology score based on percent tongue surface area covered by thrush (biofilm). Image J was used to calculate the area covered by the white plaque as well as the total dorsal surface area of each tongue in order to calculate the percentage of surface area covered. Each dot represents an individual mouse with 6 mice per group from 2 independent experiments. **(C)** Tongues were weighed, homogenized, serially diluted, and plated for *Candida* burdens in YPD agar. Log CFU counts/g of tissue are shown from n = 9 mice; bars represent mean ± SEM. *P* values of <0.05 were considered significant. Data were analyzed for statistical significance using the Graph-Pad Prism 9.0 software. Mann-Whitney test was done for statistical significance. **(D)** GMS staining highlights the abundant *Candida* hyphae and yeast (black staining; arrows) in the keratin layer of the tongue. Scale bar on 10x = 50 μm, bar on 20x = 20 μm. **(E)** The H & E staining shows the hyperplasia of the epithelium compared to the control (double-headed arrow) and epithelial invasion by *C. albicans* and with epithelial damage and infiltration by inflammatory cells (black arrows). Scale bar on 4x = 100 μm, bar on 10x = 50 μm.

To examine *Candida* infection under the immunosuppressed condition we used a previously established OPC model with some modifications. To mimic the immunosuppressed condition and to form a visible biofilm, mice were treated subcutaneously with cortisone acetate every two days starting 1 day before infection (**Figure 1A**). We treated the mice sublingually with *C. albicans* and supplied the fungus in drinking water for 3 days after infection to form a strong biofilm over the mouse tongue (**Figures 1A**, **2A**). An oral swab was taken on day -1 (before giving cortisone acetate injection) and day 0 (before infection) to determine the bacterial load. As expected, there was a heavy bacterial load after cortisone acetate injection (**Figure 1B**) when compared to before immunosuppression (on day -1). The overgrowth of bacteria in the oral cavity of immunosuppressed mice indicates the dysbiosis of oral environment and can lead to the appearance of opportunistic pathogens. Our OPC infection model showed a significant reduction in body weight of mice 3 days after *C. albicans* infection in comparison to the uninfected cortisone acetate group (**Figure 1C**). This reduction in body weight correlated with the oral biofilm that was formed over the tongue and its dissemination to the other organs of the mouse.

### 3.2 *C. albicans* forms a robust biofilm on the tongue of immunosuppressed mice

Immunosuppressed C57BL/6 mice with *C. albicans* infection developed robust biofilms over the tongue that were visible as white plaques in contrast to the cortisone acetate alone control group mice where clear tongue surface was seen (**Figure 2A**). The tongue pathology score was as follows: Tongues with no lesions = 0, unmeasurable lesions = 1, measurable lesions covering < 25% surface area = 2, between 25–75% area = 3, and > 75% surface area = 4. Oral thrush lesions (biofilms) were scored based on the surface area affected with an average of ~68% of tongue surface affected and the pathology score was found to be between 3 to 4 in the infected mice group (**Figure 2B**). CFU analysis of the *Candida* from infected mice tongue shows high fungal burden (**Figure 2C**) in agreement with the tongue pictures showing biofilm and pathological scoring (**Figures 2A, B**). In addition to assessing the fungal burden, tongue tissue harvested from mice was processed for histopathology to visualize the fungal hyphae and epithelial integrity. Microscopic analysis of GMS-stained tissue sections revealed extensive fungal adherence and hyphal penetration of the epithelial tissue (**Figure 2D**). The H & E staining showed the epithelial hyperplasia, hyperkeratosis, and infiltration of inflammatory cells compared to the cortisone acetate only (control) group. In addition, epithelial invasion by *C. albicans* with epithelial damage was seen (**Figure 2E**).

### 3.3 *Candida* oral biofilm disseminates to the gastrointestinal tract and damages its integrity

**Figure 3:**
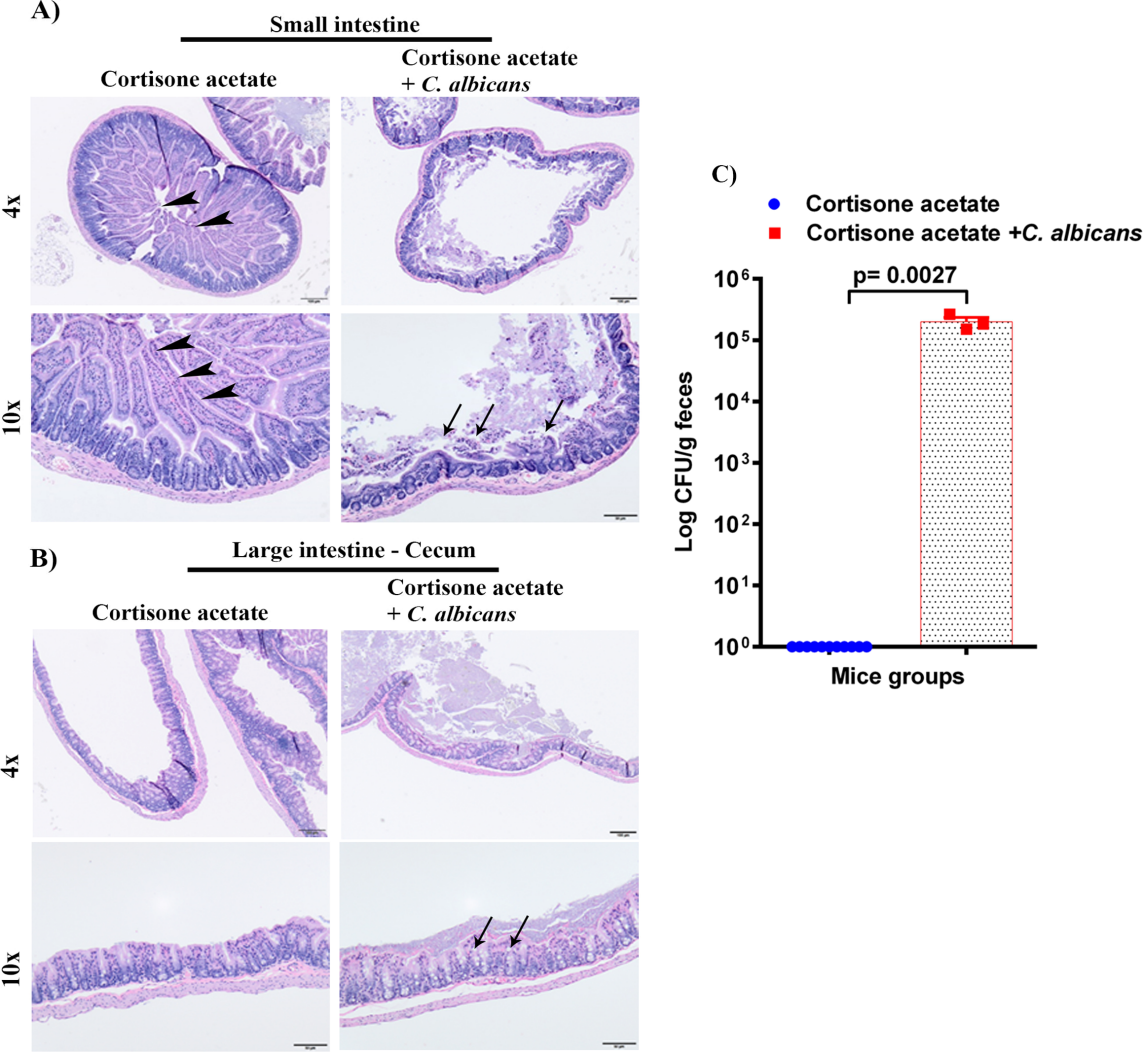
Oral *C. albicans* biofilm disseminates and causes gastrointestinal mucosal damage. The H & E stained small intestines **(A)**, and cecum **(B)** are shown. Arrowheads show normal long villi in the control small intestine. Arrows indicate mucosal damage and inflammatory cell infiltration. Bar on 4x = 100 μm, bar on 10x = 50 μm. **(C)** Stool samples were weighed, homogenized, serially diluted, and plated for *C. albicans* burdens on YPD agar. Log CFU counts/g of feces are shown from n = 11 or 3 mice; bars represent mean ± SEM. *P* values of <0.05 were considered significant. Data were analyzed for statistical significance using the Graph-Pad Prism 9.0 software. Mann-Whitney test was done for statistical significance.

To determine the dissemination of *C. albicans* after immunosuppression, we performed the histopathological analysis of GI tissues (small intestine and cecum) and CFU of stool samples. The H & E staining shows severe changes to the intestinal mucosa, with the marked attenuation of the intestinal villi in the *Candida* infected group (**Figure 3A**). To further evaluate if *C. albicans* is directly associated with intestinal villi damage, small intestine tissue sections of control and *Candida* infected groups were stained with periodic acid Schiff’s (PAS) reagent. With the present method, no fungal organisms were observed at the damaged villi (**Supplementary Figure S1**), suggesting that *C. albicans* may cause mucosal attenuation indirectly or via other unknown mechanisms, including dysbiosis in the gut microbiome. In the infected cecum sample, there were increased numbers of inflammatory cells in the mucosa (**Figure 3B**). Further, we observed a significant amount of *C. albicans* CFU in the fecal sample of the infected group (*p* < 0.0027) (**Figure 3C**).

### 3.4 Fungal dissemination from GI tract

**Figure 4:**
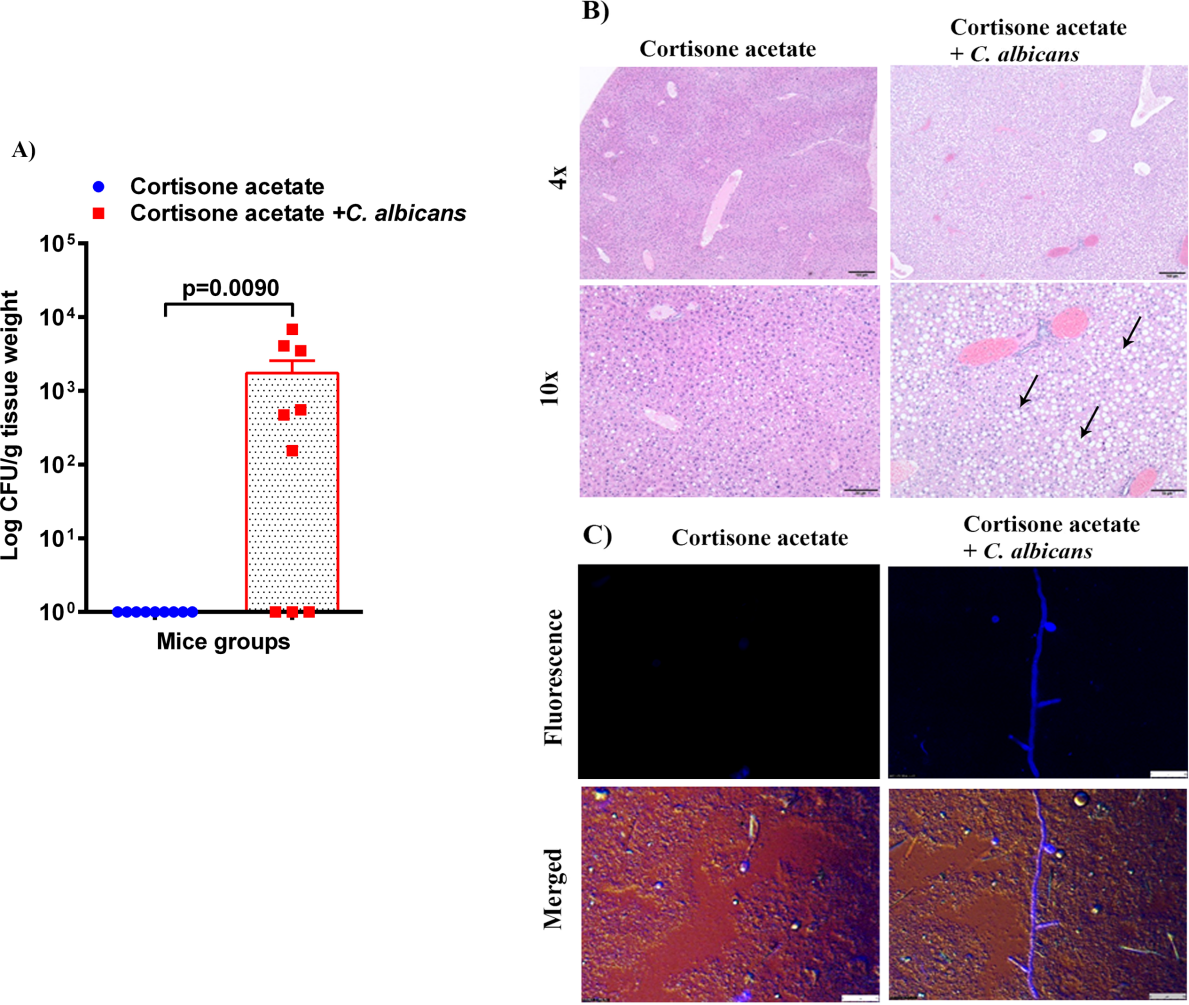
Fungal burden in the liver of immunosuppressed mice. **(A)** Liver samples were weighed, homogenized, serially diluted, and plated for *Candida* burdens on YPD agar. Log CFU counts/g of tissue are shown from n = 9 mice; bars represent mean ± SEM. *P* values of <0.05 were considered significant. Data were analyzed for statistical differences using the Graph-Pad Prism 9.0 software, and the Mann-Whitney test was done for statistical significance. **(B)** The H & E stained liver sections show increased lipid (clear circles indicated by black arrows) in the *C. albicans* affected liver, Bar on 4x = 100 μm, bar on 10x = 50 μm. **(C)** *C. albicans* hyphae was seen in infected liver homogenate stained with calcofluor white and imaged under a fluorescent microscope. Bar 25 μm.

**Figure 5:**
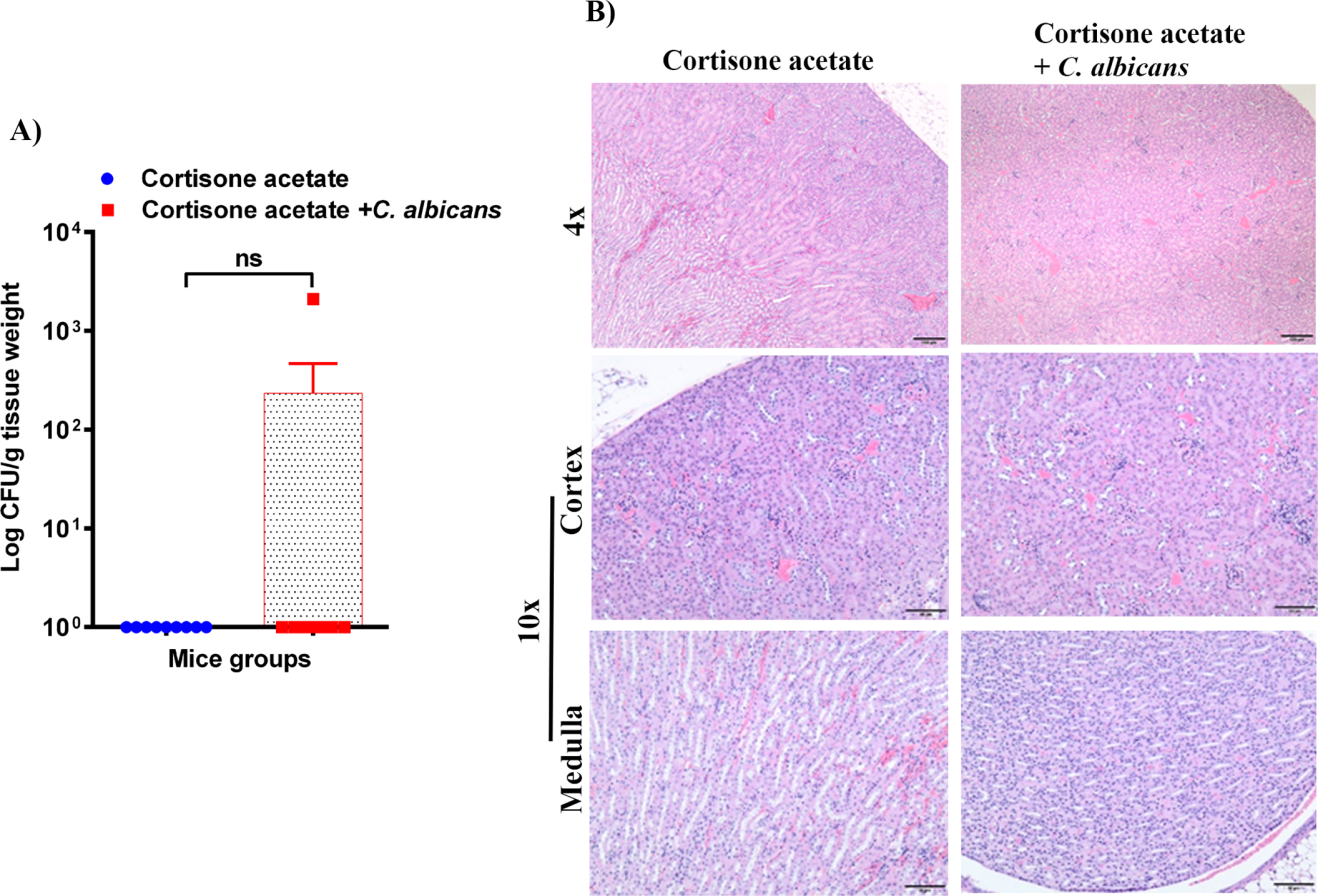
Fungal burden in the kidney of immunosuppressed mice. **(A)** Kidney samples were weighed, homogenized, serially diluted, and plated on YPD agar to determine *C. albicans* burdens. Log CFU counts/g of tissue are shown (n = 9 mice); bars represent mean ± SEM. NS, not significant. Data were analyzed for statistical differences using the Graph-Pad Prism 9.0 software, and the Mann-Whitney test was done for statistical significance. **(B)** The H & E stained kidney sections showed no difference between kidneys in control and infected groups, Bar on 4x = 100 μm; bar on 10x = 50 μm.

To assess whether the loss of GI tract integrity leads to dissemination of *C. albicans*, we performed CFU and histology analysis of the liver and kidney. We found a detectable fungal burden in the liver by determining CFU (**Figure 4A**) but not in the kidney except in one mouse (**Figure 5A**). Even though some mice did not show a fungal burden in the liver, most showed the presence of *C. albicans* with significantly higher values than controls (**Figure 4A**). These data suggest that immunosuppression and additional fungal load in the drinking water could create a favorable environment for bacterial dysbiosis and dissemination of *C. albicans*. Fungal dissemination in the liver was further assessed by H & E staining of the tissue sections and calcofluor staining of the liver homogenate. While there was no major change in histology sections of the infected liver except increased lipid vacuolation, fungal hyphae and yeasts were observed by calcofluor staining (**Figures 4B, C**). In agreement with the CFU results, histology staining showed that *C. albicans* did not disseminate to the kidney (**Figure 5B**).

### 3.5 *Enterococcus* detection following immunosuppression of mice

**Figure 6:**
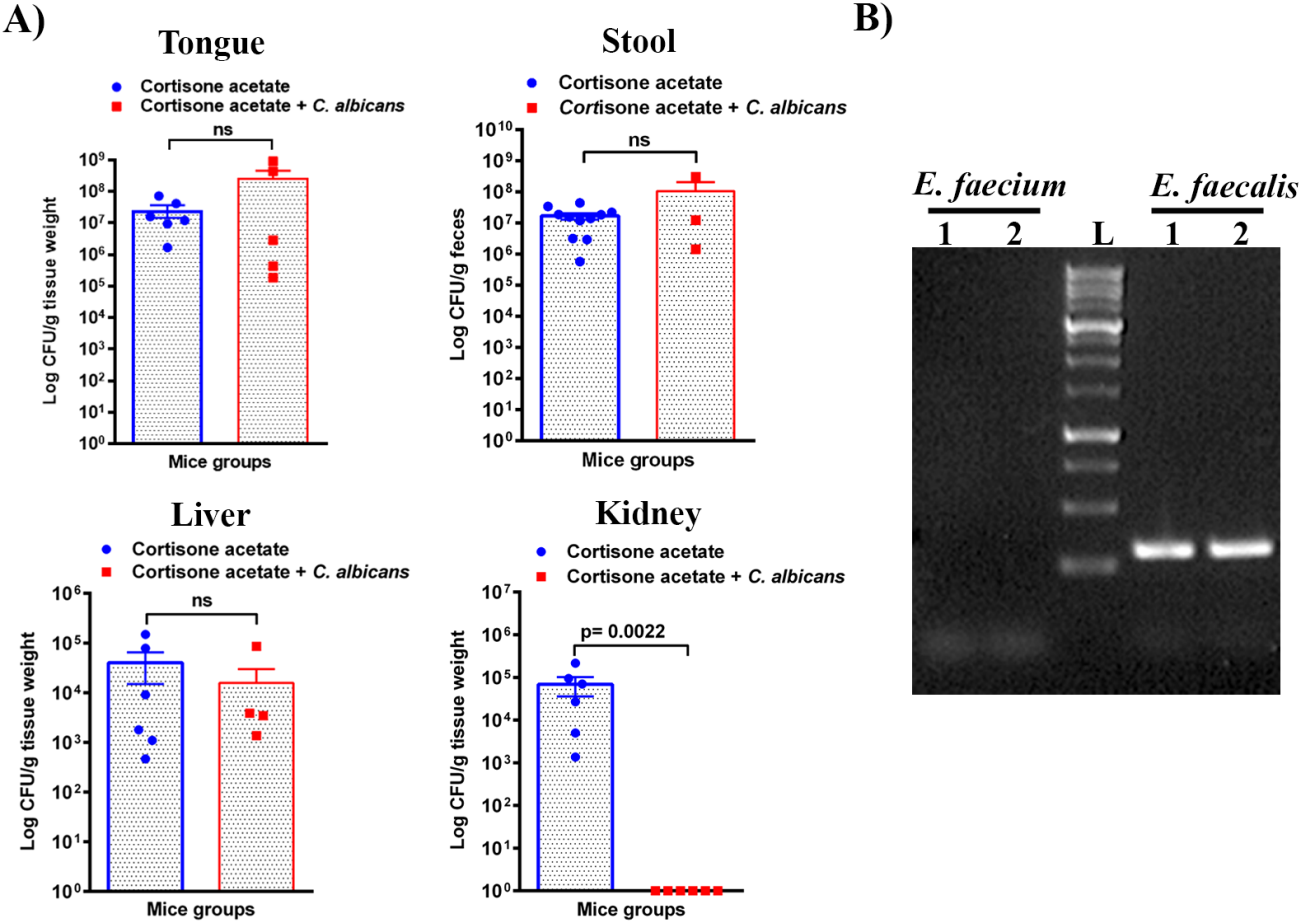
*Enterococcus* burden during *Candida* infection in immunosuppressed mice. **(A)** Tissue or stool samples were weighed, homogenized, serially diluted, and plated for *Enterococcus* growth on bile esculin (BE) agar. Log CFU counts/g of tissue or feces are shown from n = 11 or 6 or 4 mice; bars represent mean ± SEM. *P* values of <0.05 were considered significant. NS, not significant. Data were analyzed for statistical differences using the Graph-Pad Prism 9.0 software, and the Mann-Whitney test was done for statistical significance. **(B)** Identification of *Enterococcus* species by PCR for 16S rRNA gene amplification and DNA sequencing. Oligonucleotides that are specific for *E. faecalis* and *E. faecium* 16S rRNA were used (see Materials and Methods for details). Among several independent colonies from the tongue sample (also see **Figure 1B**, bile esculin agar selection) used for PCR showed positive results for *E. faecalis* and none for *E. faecium*. PCR results from two representative colonies are shown. Amplification of a 310 bp product, between 250-500 bp of the 1 kb DNA ladder, for *E. faecalis* is shown. The **Supplementary Material** includes an image of the original DNA gel as an uncropped gel image. The BLAST (NCBI) analysis of the DNA sequence of a PCR product is shown in **Supplementary Figure S4**.

*Enterococcus* sp. is one of the members of the Enterococcaceae family found in the oral cavity and GI tracts of animals and humans, and it is known to produce a plethora of virulence factors and cause disease under immunosuppressed conditions (Jett et al., 1994). Since we found an increased load of bacterial colonies from the oral swabs of the immunosuppressed mice (**Figure 1B**), we performed CFU analysis to study the dynamics of oral and intestinal enterococcal populations in response to *C. albicans* infection in immunosuppressed mice. We used BHI, BHI + NaCl, & BE agar plates. We used BE & salt agar media for confirmation (**Supplementary Figure S2**). We calculated the % CFU between BHI vs BHI + NaCl agar plates to determine *Enterococcus* sp. precisely. After *C. albicans* infection, bacterial CFU counts of BHI vs BHI + NaCl agar plates found about 25% of *Enterococcus* sp. in the tongues of *C. albicans* infected and 35% in the uninfected groups. CFU counts of the stool samples found 46% *Enterococcus* sp. in *Candida* infected and 41% in uninfected mice stools. Interestingly, *Enterococcus* sp. was absent in the kidney tissues of the fungus-infected group, while it was present in the cortisone acetate alone control group (**Figure 6A**). The absence of *Enterococcus* sp. and *C. albicans* (except one mouse) in the infection group agrees with the normal kidney histology observed (**Figure 5B**), which may suggest that synergistic fungus-bacterial interaction or high fungal burden is required for kidney tissue damage. However, further evidence is needed to support this statement. To determine the identification of *Enterococcus* species, we performed species-specific 16S rRNA PCR for *Enterococcus* species from the tongue, liver, kidney (one mouse), and stool samples (**Supplementary Figure S3**). The PCR and sequencing results from the tongue sample identified *E. faecalis*, not *E. faecium* (**Figure 6B**, **Supplementary Figure S4**).

### 3.6 Immunosuppression perturbs microbial diversity and causes dysbiosis

**Figure 7:**
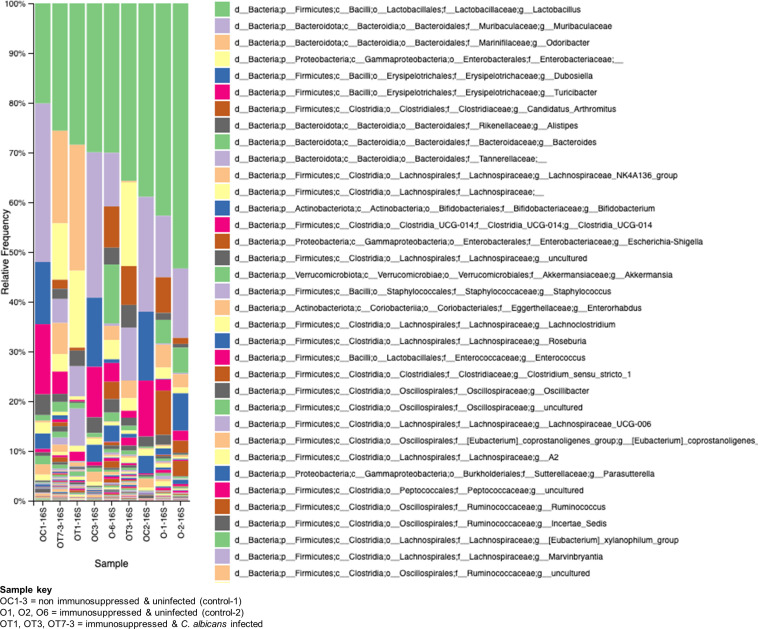
Changes in the bacterial communities in the mice gut due to immunosuppression and *C. albicans* oral biofilm. Metagenomic analysis of 16S rRNA gene amplicons of bacterial communities from immunosuppressed and *C. albicans* infected (sample keys, OT1, OT3, & OT7), immunosuppressed only control (O1, O2, & O6), and normal control (OC1-3, non-immunosuppressed & uninfected) mice fecal samples show a reversal of the beneficial bacterial members (e.g., Muribaculaceae-purple, and Erysipelotrichaceae (*Dubosiella*)-blue & magenta, etc.), of the OC groups with members of pathogenic bacteria (e.g., Enterobacteriaceae-light yellow, and Clostridiaceae (*Candidatus_arthromitus*)-rusty color, etc.), in the OT and moderately in the O groups. The key boxes read are from the top on the right panel. *Enterococcus* of Enterococcaceae (order Lactobacillales) increased ~100-fold and ~10-fold in the OT and O groups, respectively, compared to the OC group [third magenta box in the right panel & the amplicon read counts (not included)].

The GI tract harbors diverse microbes with a wide array of functions for hosts, such as protecting from pathogen invasion, maintaining a healthy gut epithelial barrier, and aiding in nutrient availability (Barko et al., 2018). The observed damages to the gut epithelial barrier and the spread of *Enterococcus* sp. and *C. albicans* to various organs of the mice in this study led us to investigate the alterations in the gut microbial communities. We used fecal samples from three groups of mice, at least three samples for each group, for metagenomic analysis. The bacterial 16S rRNA and fungal ITS2 genes were amplified from the fecal microbial DNA and sequenced on an Illumina platform. The results shown in **Figure 7** reveal major changes in bacterial communities. *Lactobacillus* is found to be high in all the groups with variable percentages. In non-immunosuppressed and uninfected control (OC group) mice, a high number of bacterial members are found to be *Lactobacillus* and Muribaculaceae, followed by *Dubosiella*, *Turicibacter*, *Alistipes*, Lachnospirales, *Bifidobacterium*, *Enterorhabdus* etc. Similar to the OC group, the immunosuppressed and uninfected control (O group) also shows abundant presence of *Lactobacillus* and Muribaculaceae followed by *Candidatus arthromitus*, *Alistipes*, *Bacteroides*, Lachnospirales, Clostridia and Enterobacteriaceae (*Escherichia_Shigella*, etc.). Remarkably, the taxonomic order of bacterial members was changed in immunosuppressed and *C. albicans* infected (OT group) and as follows: *Lactobacillus*, *Odoribacter*, Enterobacteriaceae, *Candidatus arthromitus*, *Alistipes*, Tannerellaceae, Lachnospirales, and Clostridia, etc. Overall, the members of the Muribaculaceae, Erysipelotrichaceae (*Dubosiella*), and Bifidobacteriaceae, and *Turicibacter* are considered beneficial (Liu et al., 2023; Lynch et al., 2023), and they are high numbers in non-immunosuppressed and uninfected control (OC group) mice (**Figure 7**). These bacterial members are absent in the OT or less in the O (immunosuppressed) groups, and instead, the pathogenic bacteria, including bacteria from Enterobacteriaceae, Clostridiaceae, *Odoribacter*, Tannerellaceae, and Enterococcaceae (*Enterococcus*) dominate (**Figure 7**).

**Figure 8:**
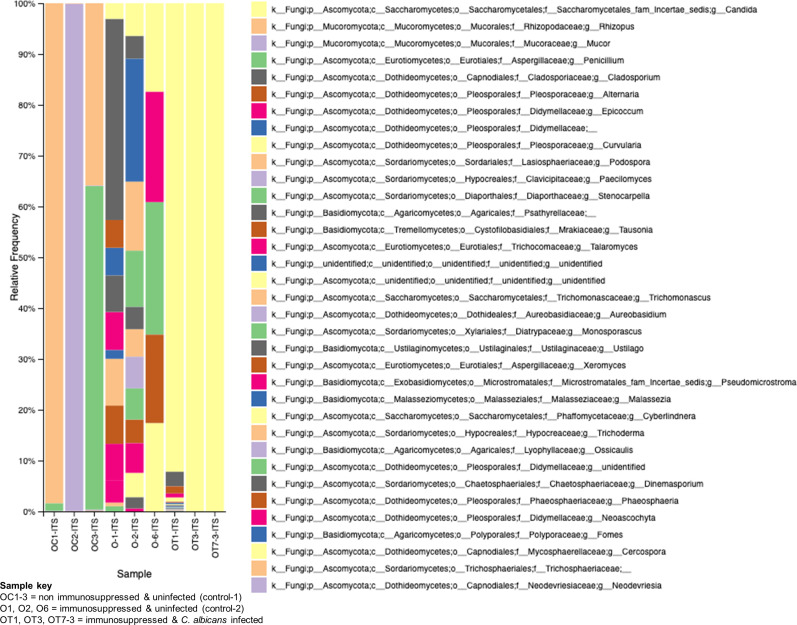
Changes in the fungal communities in the mice gut due to immunosuppression and *C. albicans* oral biofilm. Metagenomic analysis of ITS2 gene amplicons of fungal communities from immunosuppressed and *C. albicans* infected (sample keys, OT1, OT3, & OT7), immunosuppressed only control (O1, O2, & O6), and normal control (OC1-3, non-immunosuppressed & uninfected) mice fecal samples and visualized by Qiime2 software. Color coded boxes on the right panel indicates the fungal taxonomic groups.

The role of fungal communities in gut-microbial activity is poorly known. To determine the fungal taxa in the GI tracts of the above three groups of mice, we used ITS2-targeted amplicon sequencing, and the results are shown in **Figure 8**. While the non-immunosuppressed control mice (OC) group shows saprophytic fungi (*Rhizopus, Mucor*, *Penicillium*) abundantly, the immunosuppressed mice (O) group shows a mixture of fungi, including opportunistic pathogens (*Cladosporium, Alternaria*, *Epicoccum*, etc.). A small amount of Saccharomycetales yeasts, which are not *C. albicans* but likely non-albicans sp., such as *C. tropicalis* (Iliev et al., 2012), are present in these mice. Observing the OT group, we see an overwhelming presence of *Candida*, making it difficult to detect other fungal species (**Figure 8**). Although *C. albicans* is not a natural commensal or pathogen of mice (Savage and Dubos, 1967), the fungus can colonize and cause candidiasis in antibiotic-treated, neonatal, or immunosuppressed mice (Koh, 2013), suggesting bacterial communities play an important role in *C. albicans* disease.

## 4 Discussion

Opportunistic infections caused by *C. albicans* are an increasing cause of morbidity and mortality worldwide. Health conditions that include arthritis, asthma, allergic reactions, and autoimmune diseases such as lupus, sarcoidosis, or inflammatory bowel disease treated with medications like corticosteroids may weaken the host immune system and put people at risk for opportunistic infections (Ali et al., 2013). Additionally, *C. albicans* can cause severe infections in immunocompromised patients, including those with thymic aplasia, Job’s syndrome, chronic mucocutaneous candidiasis syndromes, Sjogren’s syndrome, graft-versus-host disease, HIV, leukemia, COVID-19, and others (Vila et al., 2020).

There are few OPC animal models for studying disease severity (Solis and Filler, 2012; Kong et al., 2015; Kobayashi-Sakamoto et al., 2018). Similar to Solis and Filler’s model, our animal model includes cortisone acetate every other day to maintain the immunosuppression condition. In addition, we added an extra criterion of administering *C. albicans* in drinking water for three days to mimic the high oral *Candida* burden reported in certain groups of patients with underlying health conditions (e.g., cancer and during radio- or chemotherapy (Kauffman, 2011; Jain et al., 2016)) (**Figure 1A**). These patients will likely swallow their saliva, passing through the GI tract, and *C. albicans* can cause disease under immunosuppressive conditions in conjunction with the resident microbiome.

High levels of fungal biofilm over the surface of the tongue increases virulence and shows high resistance to various antimicrobials and host immune mechanisms (Vila et al., 2020). Our OPC model was found to work efficiently as evident by thick biofilm over most of the surface of the tongue (**Figure 2A-C**) and reduction in body weight (**Figure 1C**). The importance of morphological conversion from yeast to hyphae in mucosal invasive infections is well studied (Phan et al., 2000; Kong et al., 2015; Swidergall et al., 2021). GMS and histological staining of tongue sections clearly showed hyphae where the fungus invaded and damaged the epithelial layer (**Figure 2D, E**). Following tissue damage, immune cells are the first line of defense in the fungal killing and tissue repair process (Gazendam et al., 2014) and we observed infiltration of inflammatory cells (mostly neutrophils based on their morphology) in infected tongue tissues (**Figure 2E**).

As in the oral cavity, *C. albicans* is a normal microbial member of the GI tract in healthy humans (MacCallum, 2012; Mason et al., 2012). During immunosuppression, dysbiosis of the microbiome can augment the proliferation of pathogenic microbes (**Figures 7**, **8**) and can cause life-threatening illnesses. In our OPC model, as we infected the mice with *C. albicans* through the oral route, we analyzed the histology of the GI tract. The morphology of the small and large intestine was found to be compromised, and more inflammatory cell infiltration was observed (**Figure 3A, B**). *C. albicans* has evolved to effectively colonize certain mucosal surfaces in human hosts. This colonization is typically commensal with the normal host microbiome, but when the microbiome is altered due to immunosuppression and overgrowth of *C. albicans*, there can be direct or indirect damage to the mucosal tissues (Vila et al., 2020; d’Enfert et al., 2021). Based on our results of PAS staining of the small intestine, *C. albicans* may be involved in causing mucosal damage indirectly (**Supplementary Figure S4**), probably through its cytolytic peptide toxin (candidalysin) produced from the fungal hyphal growth (Moyes et al., 2016) or via a combination of bacterial virulence factors. After the loss of mucosal barrier integrity in the GI tract, the fungal and bacterial cells can disseminate and colonize non-GI organs. It is interesting to determine if non-toxic small molecules inhibiting *C. albicans* hyphal growth (Vediyappan et al., 2013; Veerapandian and Vediyappan, 2019) could prevent the bacterial dysbiosis and or candidalysin-mediated host cell damage. CFU analysis of the liver and kidney showed that *C. albicans* and *Enterococcus* were present in the liver but not (*C. albicans*) in the kidney (**Figures 4**–**6**). This was further supported by calcofluor staining (**Figure 4C**). Interestingly, *C. albicans* did not colonize the kidney in the present study (except one mouse, **Figure 5A**). In support of our data, a previous study has reported the absence of fungal colonization in kidney samples. However, during coinfection with *S. aureus*, *Candida* was seen (Kong et al., 2015).

In addition to immunosuppression and fungal infection, the other major factor responsible for the severity of infection is dysbiosis of the host microbiome. It is now well accepted that the bacterial component plays a major role in the severity of local and systemic candidiasis (Harriott and Noverr, 2011; Sultan et al., 2018; Bertolini et al., 2019). In clinical settings, the use of antibiotics is the major reason behind dysbiosis in the oral cavity and GI tract. This alters the polymicrobial interactions, which may impact *C. albicans’* growth positively or negatively depending on the surrounding environment. In our model (**Figure 1A**), we excluded the use of antibiotics because we wanted to characterize the role of immunosuppressants in causing dysbiosis. Interestingly, cortisone acetate immunosuppression alone causes bacterial dysbiosis where 35% and about 41% of the bacterial population is found to be *Enterococcus* spp. in the tongue and stool samples, respectively, as confirmed by its differential growth on bile esculin selective agar and on salt agar medium (BHI agar + 6.5% NaCl) (**Figures 1B**, **Supplementary Figure S2**). The identification of *E. faecalis* was confirmed by PCR amplification of the 16S rRNA gene and its DNA sequence analysis (**Figures 6B**, **Supplementary Figures S3**, **S4**). Further, metagenomic analysis of the fecal microbiome confirmed the alteration of the bacterial and fungal diversity (**Figures 7**, **8**). Particularly, the beneficial bacteria (*Bifidobacterium, Dubosiella*, *Turicibacter*, etc.) are completely absent in the immunosuppressed and *C. albicans* infected mice group (OT) and immunosuppressed and uninfected control (O group) when compared to the normal control (OC) mice group. Instead, pathogenic bacteria, such as *Clostridia, E. coli*, and *Enterococcus* were represented. *Enterococcus* is a member of the ESKAPE group that contains *Enterococcus faecium*, *Staphylococcus aureus*, *Klebsiella pneumoniae*, *Acinetobacter baumannii*, *Pseudomonas aeruginosa*, and *Enterobacter* species, known for their high virulence and antimicrobial resistance.

Bifidobacteria has proven to be a major antagonist against *C. albicans* (Mirhakkak et al., 2021; Ricci et al., 2022). The *Turicibacter* genus is widespread in the gut microbiota of mice, humans, cows, pigs, and chickens (Lynch et al., 2023), and recently it has been shown to protect mice from severe *Citrobacter rodentium* infection (Hoek et al., 2023). Interestingly, the absence of Turicibacteriales in the intestinal microbiota is closely linked to an increased susceptibility to *C. rodentium.* Similarly, infection with *C. albicans* in immunosuppressed mice inhibits saprophytic fungi like *Rhizopus, Mucor*, *Penicillium*, etc. Typically, *C. albicans* can alter the gut microbiome through direct mechanisms, such as competing for niches and nutrients or secreting metabolites that inhibit the growth of host microbes. Zhai et al. demonstrated by sequencing and comparative analysis of the mycobiota of fecal and blood samples from hematopoietic cell transplant (HCT) patients that the involvement of a complex dynamic translocation of *Candida* species in the bloodstream and their intestinal expansion was associated with a substantial loss of bacterial burden and diversity (anaerobes) (Zhai et al., 2020). Studies on gut fungal diversity after *C. albicans* infection are limited, and our studies pave the way for more understanding of gut fungal microbiome in different groups of mice like non-immunosuppressed (OC) or immunosuppressed (O) or immunosuppressed and *C. albicans* infected (OT). The difference between O and OT groups clearly shows that the overgrowth of *C. albicans* in the gut inhibits other fungi present in the mouse gut microbiome (Wang et al., 2024). Further, oral *Candida* infection under immunosuppressed conditions leads to dysbiosis of bacteria and fungi, particularly the beneficial bacteria were replaced with pathogenic bacterial groups, and depletion of several saprophytic fungi.

**Figure 9:**
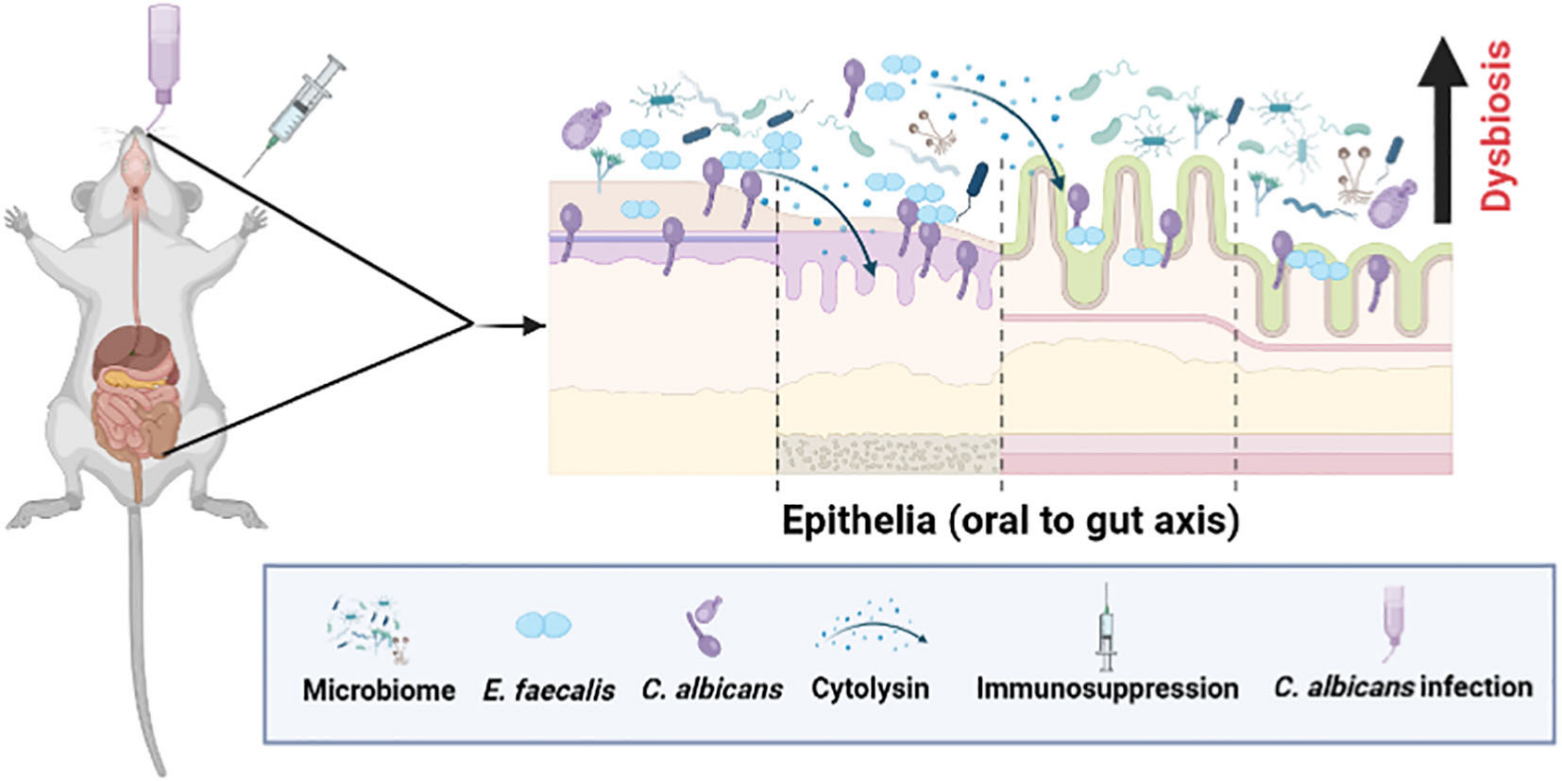
A schematic showing microbial dysbiosis in an immunosuppressed mouse model of oral candidiasis. Under immunosuppressed conditions, orally infected *C. albicans* and altered oral microflora, including *Enterococcus faecalis*, overgrow in the oral cavity and cause infections. These ingested oral and altered gut microbes can colonize, and compromise the mucosal epithelial barrier and translocate to the internal organs. Physical interaction between *C. albicans* and *E. faecalis* promotes their accumulation on host cells, and augments host cell damage through cytolysin from certain strains of *E. faecalis* (Bertolini et al., 2019; Kapitan et al., 2024) (see text for details). This figure was created using Biorender.

*Enterococcus* sp. is widely reported to coexist as an antagonist or synergist with *C. albicans* in human infected samples (Hermann et al., 1999; Mason et al., 2012; Garsin and Lorenz, 2013; Kovac et al., 2013; Graham et al., 2017). Using an organotypic oral epithelial model, a recent study has shown the synergistic interaction between *C. albicans* and *E. faecalis* (Krishnamoorthy et al., 2020). *E. faecalis* is reported to degrade the epithelial junction protein E-cadherin and help *C. albicans* to invade in an *in vivo* OPC infection model (Bertolini et al., 2019). A recent study has shown that the cell wall components (peptidoglycan subunits) of bacteria promoted filamentation and invasive infection of *C. albicans* in the GI tract (Tan et al., 2021). The enhanced growth of *E. faecalis* observed in our study following immunosuppression may account for the oral and GI-mucosal barrier damages and dissemination of *C. albicans*. A schematic in **Figure 9** shows how the alteration of microflora due to immunosuppression in this mouse model of oral candidiasis affects the mucosal barrier and promotes the colonization and dissemination of *C. albicans.* Based on our results and several recent studies (Mason et al., 2012; Koh, 2013; Bertolini et al., 2019; Kapitan et al., 2024), physical interaction between *C. albicans* and bacteria (e.g., *E. faecalis*) promotes their accumulation on host mucosal cells, and causes host cell damage through bacterial or fungal or both of their hydrolytic enzymes or toxins. Since BE and BHI + NaCl agar media are selective and differential growth media for isolating and identifying members of *Enterococcus*, our study is biased toward this group of bacteria. Our results also show the role of pathogenic Enterobacteriaceae members as they replaced the beneficial members, such as Muribaculaceae and Erysipelotrichaceae (*Dubosiella*) of the OC (normal control) groups, with *Klebsiella* and other enteric Gram-negative members (**Figure 7**). Niemiec et al. have shown that *Proteus mirabilis*, an Enterobacteriaceae member, interacted with *C. albicans* and non-albicans species and caused increased enterocyte damage (Niemiec et al., 2022). Thus, the contribution of other bacteria to the mucosal tissue damage and dissemination of *C. albicans* can’t be ruled out, and future studies will explore this potential contribution.

## 5 Conclusion

Analysis of the mouse OPC model described in this report highlights how an oral infection of *C. albicans* can lead to its dissemination under the immunosuppressed condition in the absence of antibiotics or chemotherapeutic agents. We also found alteration of the gut microbiome under the above conditions that promote the growth of *Enterococcus* sp., *C. albicans*, and likely other pathogenic bacteria in immunosuppressed mice, which may have contributed to the severity of mucosal damage and dissemination. The coexistence of these types of endogenous opportunistic pathogens in humans is a high risk for immunocompromised patients.

## Data Availability

The datasets presented in this study can be found in online repositories. The names of the repository/repositories and accession number(s) can be found in the article/**Supplementary Material**.
