# Supplementary material for: A mouse model of immunosuppression facilitates oral Candida albicans biofilms, bacterial dysbiosis and dissemination of infection
Source: Front Cell Infect Microbiol. 2025 Jan 20;14:1467896. doi: 10.3389/fcimb.2024.1467896 (PMC11788080; doi:10.3389/fcimb.2024.1467896)
Supplement: Supplementary file 1 [file DataSheet1.pdf]

A mouse model of immunosuppression facilitates oral *Candida albicans* biofilms, bacterial dysbiosis and dissemination of infection

Raja Veerapandian[1,@,#], Anuja Paudyal[1,@,&], Sarah M. Schneider[2,$], Sonny T. M. Lee[1], and Govindsamy Vediyappan[1,2,*]

[1]Division of Biology, Kansas State University, Manhattan, KS, USA.
[2]Diagnostic Medicine and Pathobiology, Kansas State University, Manhattan, KS, USA.
Present address: [#]Department of Molecular and Translational Medicine, Texas Tech University Health Sciences Center, El Paso, TX, USA; [&]Division of Hematology, Brigham and Women's Hospital, Boston, MA, USA; [$]Department of Pathology, University of Georgia, Athens, GA, USA. @, contributed equally.

*** Correspondence:**
Corresponding Author, Govindsamy Vediyappan, gvediyap@ksu.edu

Supplementary Figure 1. The periodic acid Schiff's (PAS) stained small intestine showing damaged villi (arrows) in the immunosuppressed and *C. albicans* infected but not in the immunosuppressed and uninfected control mice group. The mucosal damage of the GI tract may be due to the indirect effect of *C. albicans* infection as no fungal organisms were identified at the damaged regions.

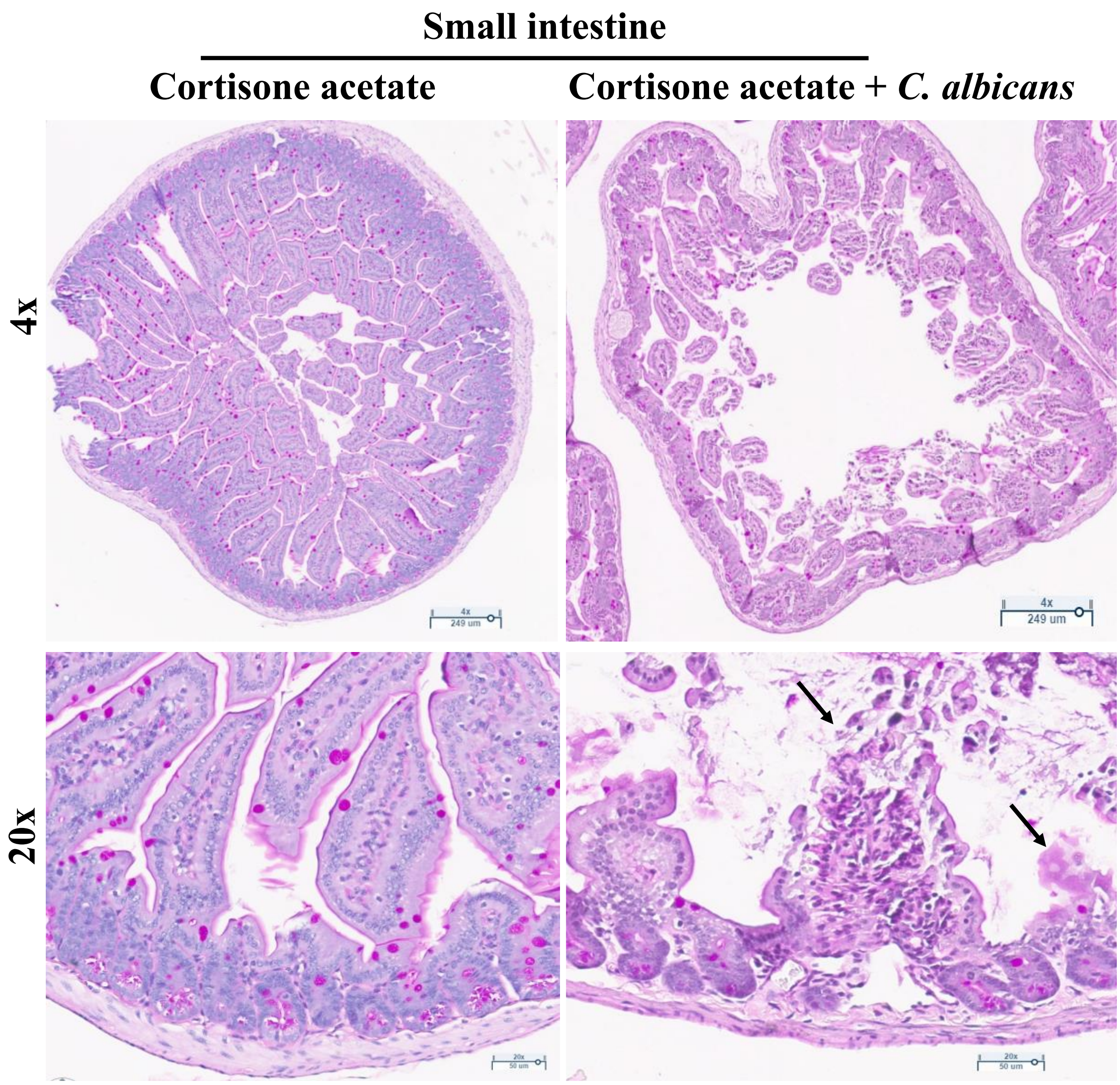

Supplementary Figure 2. Growth of microbes from stools of immunosuppressed uninfected (control) and *C. albicans* infected mice on BHI, BHI + NaCl (6.5%), and BE agar plates. Aliquots of diluted homogenate of stool samples were plated on agar plates and incubated as mentioned in the Materials and Methods. A representative plate from each medium is shown. Large colonies found on BHI, and BE in the infected group indicate *C. albicans* (arrows) and are absent on BHI + NaCl as a high concentration of NaCl is inhibitory. *Enterococci* are bile resistant and tolerant to high salt concentrations (6.5%), and hence, they can be grown on both BE and salt agar plates. The dark brownish-black color found in the BE plates is due to the reaction of BE media components with bile utilizing microbial metabolites.

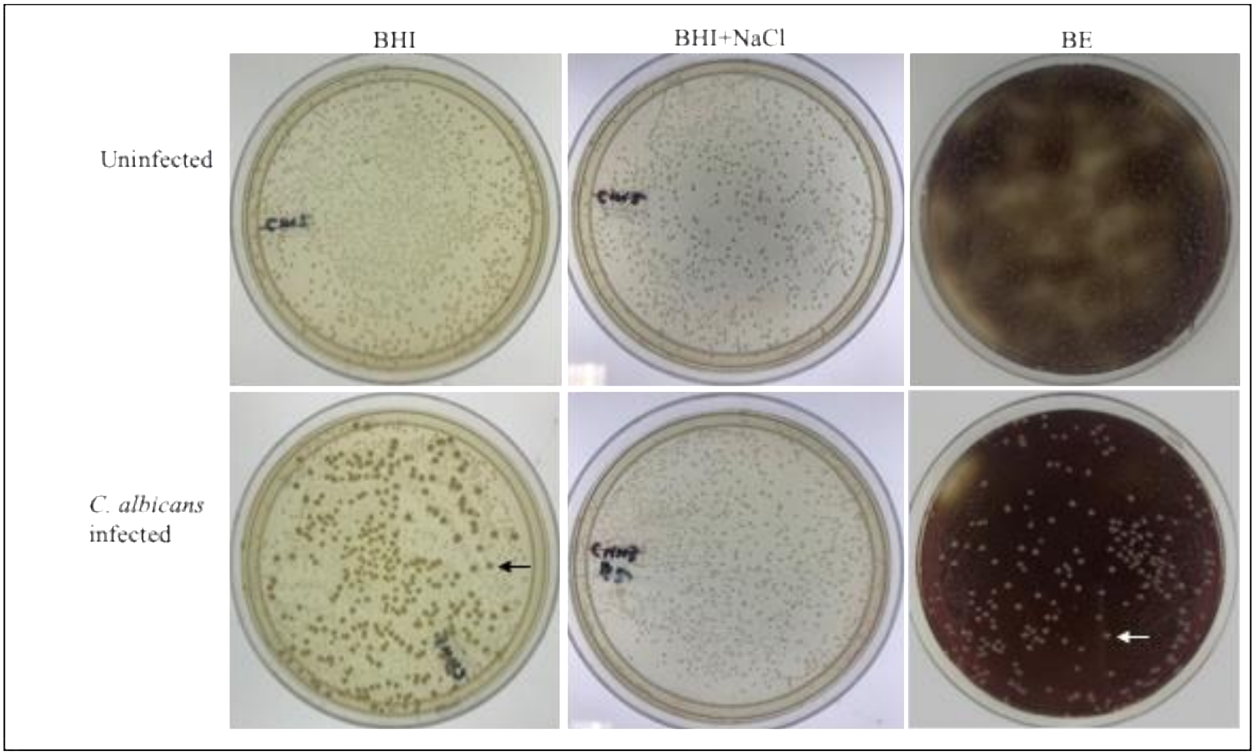

Supplementary Figure 3. Verification of *Enterococcus* sp. 16S rRNA gene by PCR method. Oligonucleotides that are specific for *E. faecalis* and *E. faecium* 16S rRNA were used as described in the Material and Methods. Two independent colonies from different tissue (tongue, liver, kidney) or stool samples of uninfected control, and *C. albicans* infected were used for PCR analysis. Colonies from BHI + NaCl or BE plates (Supplementary Figure 2, and not shown) were selected for PCR. A PCR product of 310 (arrows) was amplified with oligonucleotides specific for *E. faecalis* but not with *E. faecium*. An unknown white colony that was non-color producing on BE agar served as negative control (-ve) in the PCR.

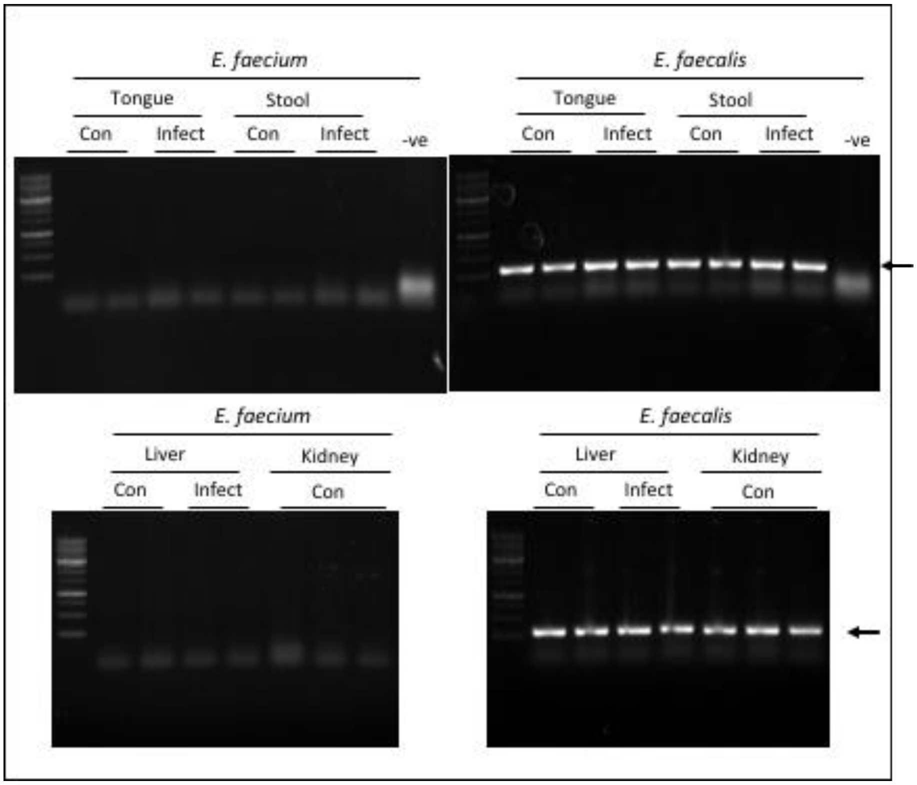

Supplement Figure 4. Comparison of *Enterococcus sp* 16S rRNA gene sequence with the NCBI database. The identified partial 16S rRNA gene sequence from *E. faecalis* was deposited in the GenBank database with accession No. MZ868593.

Enterococcus faecalis strain AMHWRB4 16S ribosomal RNA gene, partial sequence

Sequence ID: MZ672033.1 **Length:** 1421 **Number of Matches:** 1

Range 1: 167 to 439 GenBank Graphics ▼ Next Match ▲ Previous

Score	Expect	Identities	Gaps	Strand
478 bits(529)	2e-130	269/273(99%)	0/273(0%)	Plus/Plus

```
Query  1    GCTTTCGGGTGTCGCTGATGGATGGACCCGCGGTGCATTAGCTAGTTGGTGAGGTAACGG  60
            ||||||||||||||||||||||||||||||||||||||||||||||||||||||||||||
Sbjct  167  GCTTTCGGGTGTCGCTGATGGATGGACCCGCGGTGCATTAGCTAGTTGGTGAGGTAACGG  226

Query  61   CTCACCAAGGCCACGATGCATAGCCGACCTGAGAGGGTGATCGGCCACACTGGGACTGAG  120
            ||||||||||||||||||||||||||||||||||||||||||||||||||||||||||||
Sbjct  227  CTCACCAAGGCCACGATGCATAGCCGACCTGAGAGGGTGATCGGCCACACTGGGACTGAG  286

Query  121  ACACGGCCCAGACTCCTACGGGAGGCAGCAGTAGGGAATCTTCGGCAATGGACGAAAGTC  180
            ||||||||||||||||||||||||||||||||||||||||||||||||||||||||||||
Sbjct  287  ACACGGCCCAGACTCCTACGGGAGGCAGCAGTAGGGAATCTTCGGCAATGGACGAAAGTC  346

Query  181  TGACCGAGCAACGCCGCGTGAGTGAAGAAGGTTTTCGGATCGTAAAACTCTGTTGTTAGA  240
            ||||||||||||||||||||||||||||||||||||||||||||||||||||||||||||
Sbjct  347  TGACCGAGCAACGCCGCGTGAGTGAAGAAGGTTTTCGGATCGTAAAACTCTGTTGTTAGA  406

Query  241  GAAGAACAAGGACGTTAGTAANGNNACGTCCCC  273
            |||||||||||||||||||||    ||||||||
Sbjct  407  GAAGAACAAGGACGTTAGTAACTGAACGTCCCC  439
```

Query: DNA sequence of PCR product with primers specific for *E. faecalis,* sample #1

Sbjct: NCBI blast match
