# Supplementary material for: A mouse model of immunosuppression facilitates oral Candida albicans biofilms, bacterial dysbiosis and dissemination of infection
Source: Front Cell Infect Microbiol. 2025 Jan 20;14:1467896. doi: 10.3389/fcimb.2024.1467896 (PMC11788080; doi:10.3389/fcimb.2024.1467896)
Supplement: Supplementary file 2 [file DataSheet2.pdf]

A mouse model of immunosuppression facilitates oral *Candida albicans* biofilms, bacterial dysbiosis and dissemination of infection

Raja Veerapandian[1,@,#], Anuja Paudyal[1,@,&], Sarah M. Schneider[2,$], Sonny T. M. Lee[1], and Govindsamy Vediyappan[1,2,*]

[1]Division of Biology, Kansas State University, Manhattan, KS, USA.
[2]Diagnostic Medicine and Pathobiology, Kansas State University, Manhattan, KS, USA.
Present address: [#]Department of Molecular and Translational Medicine, Texas Tech University Health Sciences Center, El Paso, TX, USA; [&]Division of Hematology, Brigham and Women's Hospital, Boston, MA, USA; [$]Department of Pathology, University of Georgia, Athens, GA, USA. @, contributed equally.

*** Correspondence:**
Corresponding Author, Govindsamy Vediyappan, gvediyap@ksu.edu

Uncropped DNA gel image for Figure 6B

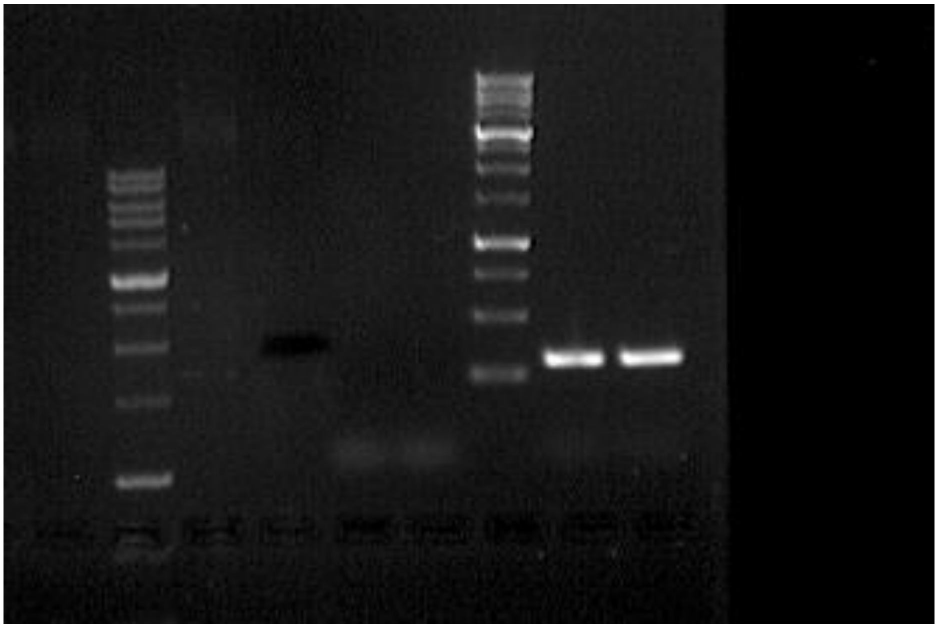

Uncropped DNA gel images for Supplementary Figure S3

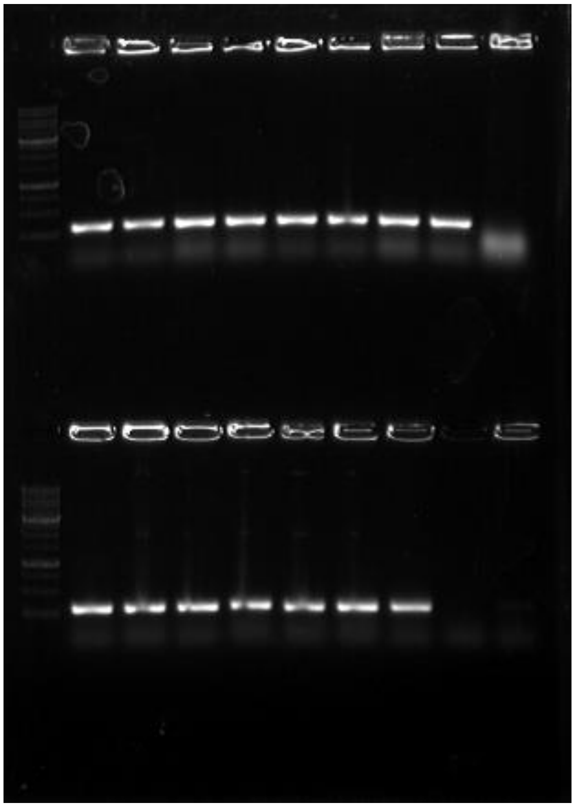

E. faecalis primers

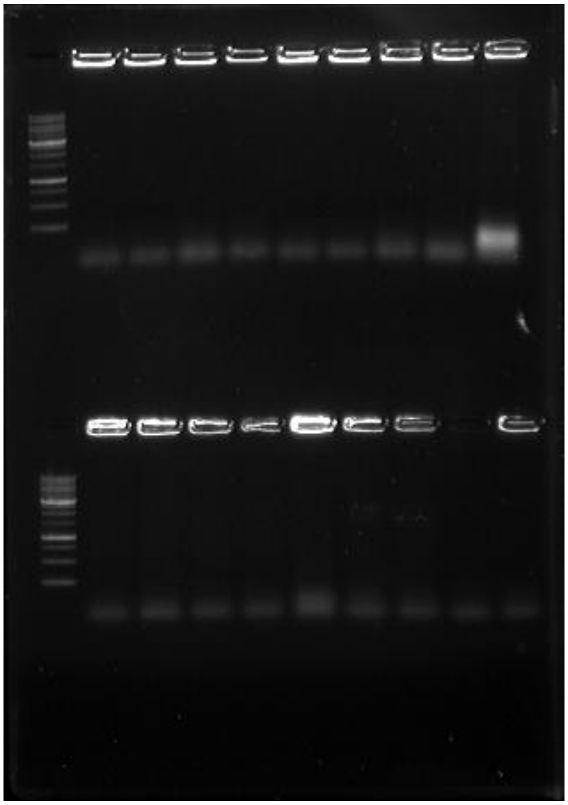

E. faecium
